# The Extraction, Anticancer Effect, Bioavailability, and Nanotechnology of Baicalin

**DOI:** 10.23937/2572-3278.1510011

**Published:** 2016-03-22

**Authors:** Ondrea A. Moore, Ying Gao, Allen Y. Chen, Ross Brittain, Yi Charlie Chen

**Affiliations:** College of Science, Technology & Mathematics, Alderson Broaddus University, Philippi, WV 26416, USA

## Abstract

The dried root of Baikal skullcap (*Scutellaria baicalensis*) has been historically and widely used in traditional Eastern medicine. Modern science proved that baicalin is the major bioactive responsible for the physiological activity of Baikal skullcap. Baicalin, a flavonoid found in several species in the genus *Scutellaria*, has been regarded as a potent anticancer agent. In this review, we present the main extraction methods, anticancer activity and bioavailability of baicalin. Besides, the utilization of nanotechnology to improve the bioavailability of baicalin is also mentioned.

## Introduction

Cancer is a group of diseases involving abnormal cell growth with the potential to invade or spread to other parts of the body. In 2016, 1,685,210 new cancer cases and 595,690 cancer deaths are projected to occur in the United States [1]. Surgery, radiation therapy, chemotherapy, and targeted therapy are the most common types of cancer treatment. However, they might cause severe side effects in patients. Fatigue, a common early and chronic side effect of irradiation, was reported in up to 80% of patients during radiation therapy and 30% of follow-up visits [2]. The incidence of chemotherapy-induced nausea and vomiting (CINV) in patients was 59.7% within 24 hours after treatment. And the incidence of delayed-CINV was 22.9% in patients who experienced neither vomiting nor nausea during the first 24 hours post-chemotherapy [3]. Thus, it is important to find an alternative cancer treatment with fewer adverse side effects.

**Figure 1:**
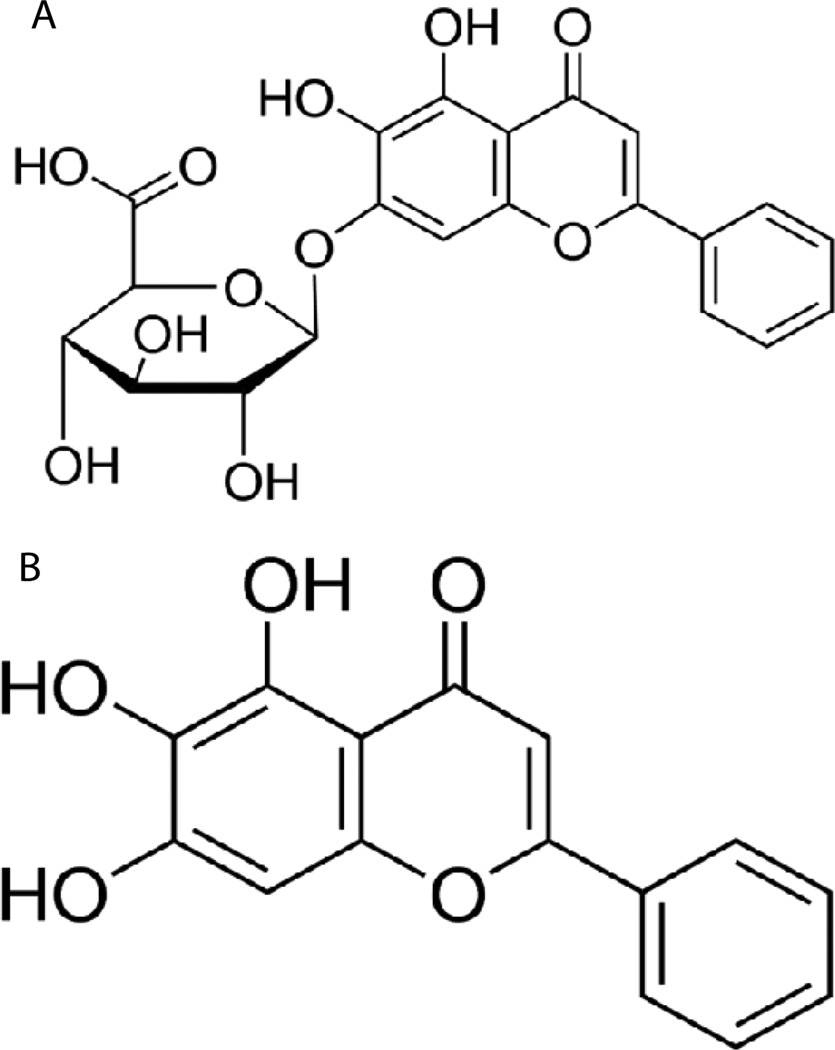
Chemical structure of baicalin (**A**) and its aglyconebaicalein (**B**).

**Figure 2:**
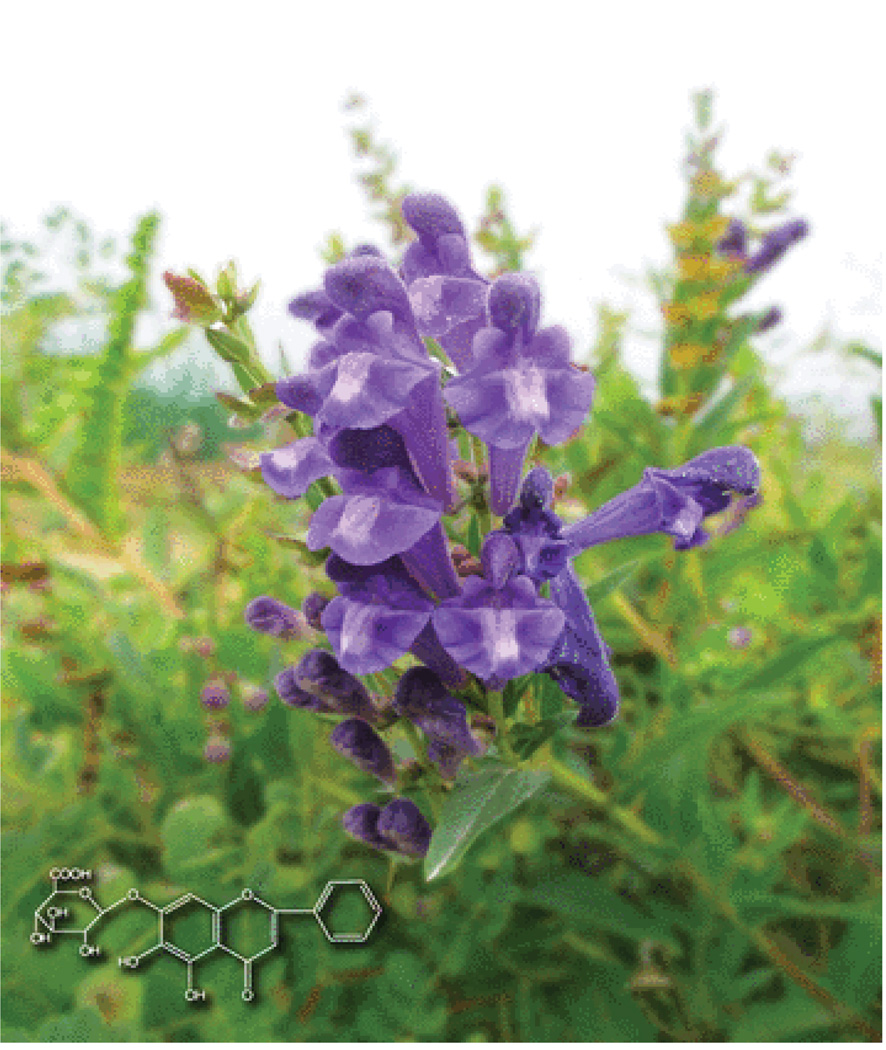
Baikal skullcap (*Scutellaria baicalensis*) [30].

There is increasing interest in selecting specific dietary flavonoids as potential anticancer agents because of their high efficiency and low toxicity [4,5]. Flavonoids are a class of plant secondary metabolites, which consist of two phenyl rings and a heterocyclic ring. Some of the plants containing flavonoids have been used in traditional Chinese medicine for thousands of years. Baicalin (Figure 1A) is a flavonoid found in the Chinese herb *Scutellaria baicalensis* (also known as Baikal skullcap) (Figure 2). Baicalein (Figure 1B) is the aglycone of baicalin. Baicalin has been applied in pharmaceuticals, food and cosmetics industries. Different extraction methods are employed to isolate baicalin from *Scutellaria baicalensis.* Among them, heat reflux extraction (HRE), ultrasound-assisted extraction (UAE) and supercritical fluid extraction (SFE) are three most widely used extraction methods. Baicalin displays activities against cancer, bacterial infections, and oxidative stress-related disease [6,7]. Nevertheless, like other flavonoids, poor bioavailability limits its application [8,9]. Strategies to improve the bioavailability of baicalin are being explored [9–11]. The nanonization of bioactives derived from herbal medicines has gained more and more attention in recent years. Nanoformulations, such as nanoparticles, nanoemulsions and liposome, are colloidal systems with particles varying in size from 10 nm to 1000 nm [12]. Nanoformulation of herbal medicines possess benefits, such as improving solubility, enhancing bioavailability, increasing absorbency of the organism, reducing medicinal herb doses, and achieving steady-state therapeutic levels of drugs over an extended period compared with traditional herbal medicine preparations [12]. Though nanotechnology shows a lot of promise, further studies should be conducted to validate this [13].

In this review, the extraction, anticancer activity, bioavailability, and the application of nanotechnology to increase the bioavailability of baicalin are described.

## Extraction

**Figure 3:**
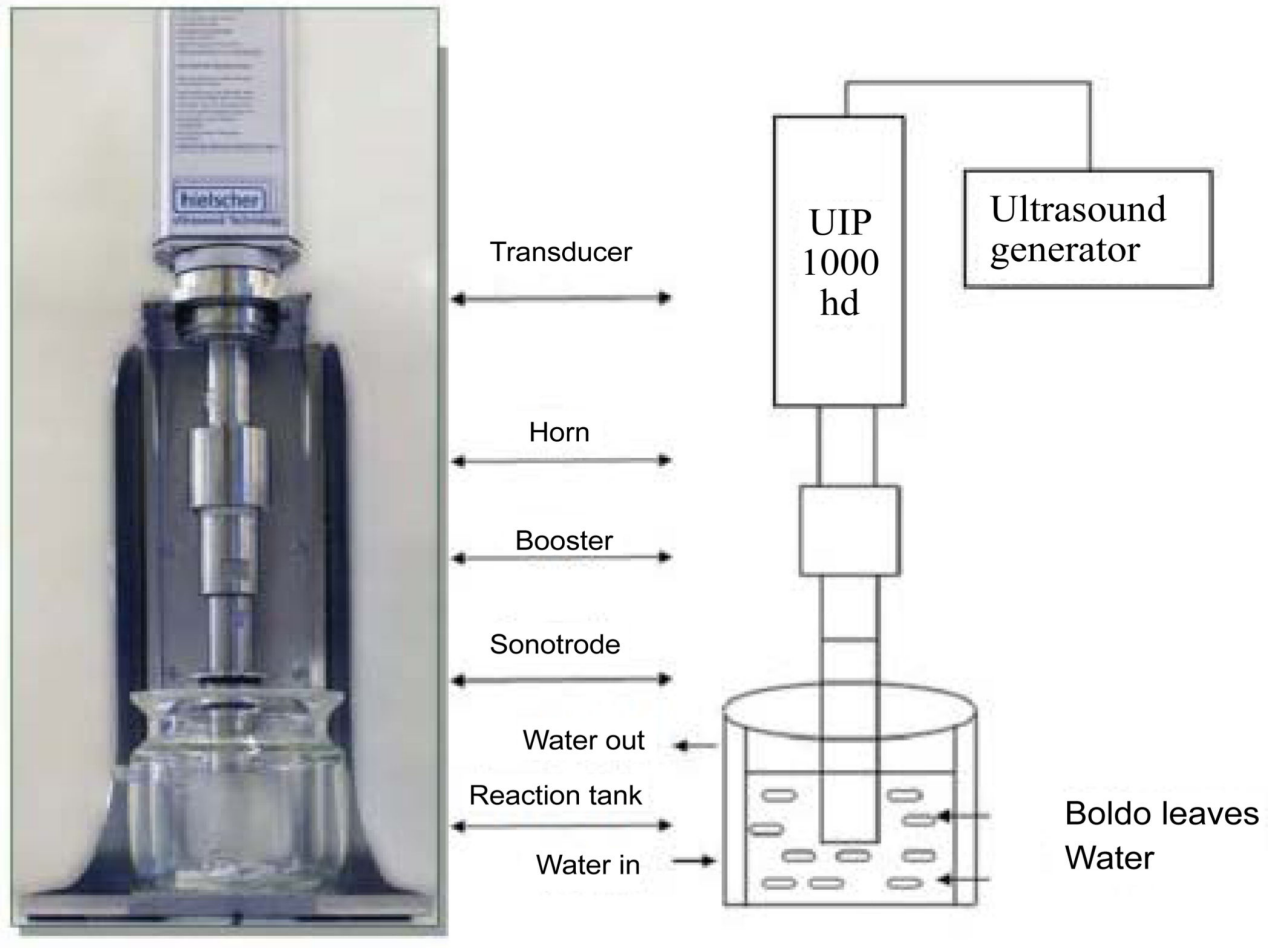
Ultrasound-assisted extraction (UAE) method [31].

**Figure 4:**
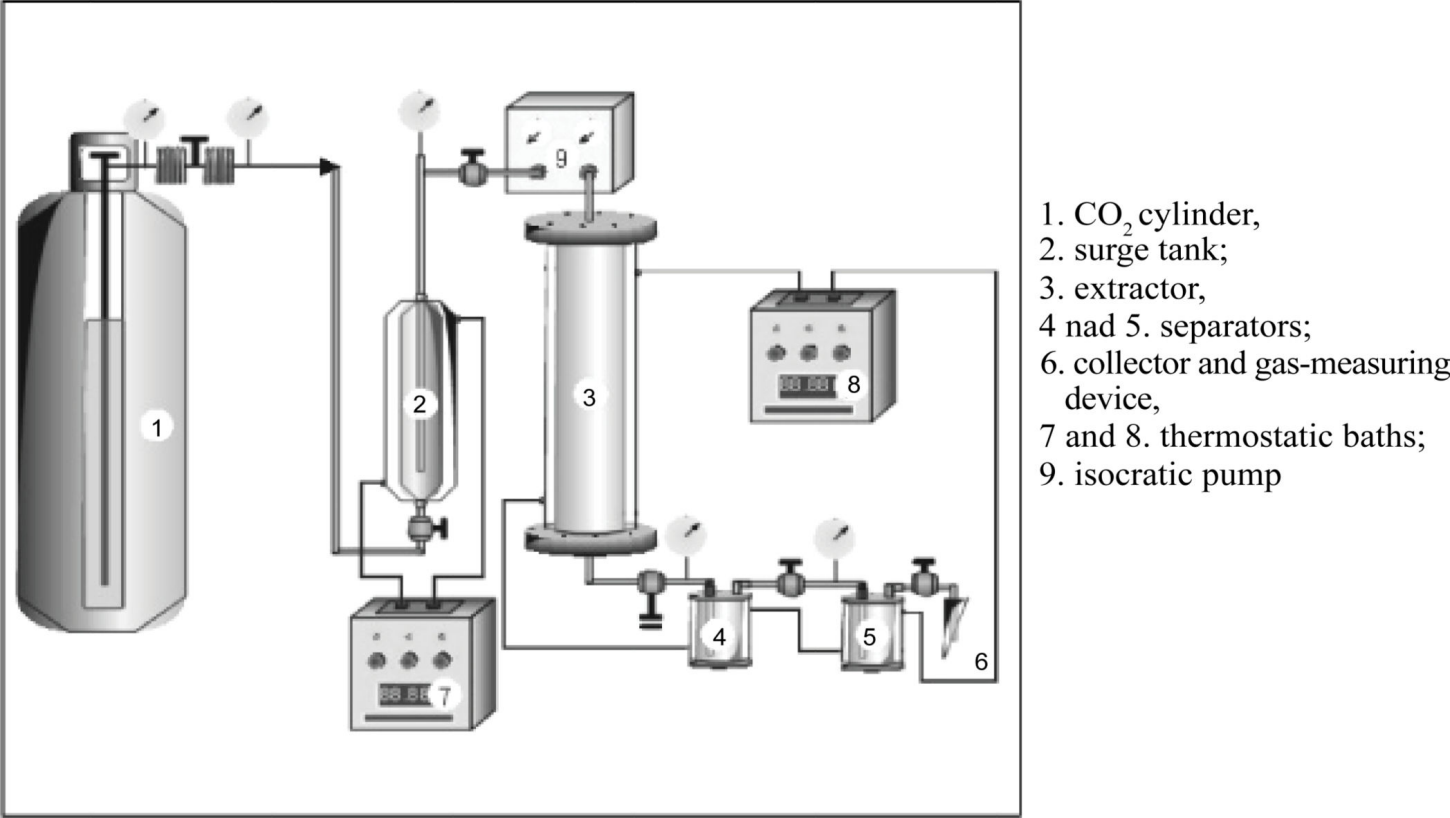
Supercritical fluid extraction (SFE) method [32].

Heat reflux extraction (HRE), ultrasound-assisted extraction (UAE) (Figure 3), and supercritical fluid extraction (SFE) (Figure 4) are common extraction methods to isolate and purify baicalin. HRE is a conventional extraction method which is versatile but time-consuming. UAE works through the process known as cavitation which forms small bubbles in liquids and the mechanical erosion of solid particles. This causes a local increase in temperature and pressure which favor solubility, diffusivity, penetration and transport. Compared with HRE, UAE reduced the extraction time, the extraction temperature, and the solvent consumption. UAE is considered as an economical alternative to replace traditional extraction methods [14]. Previous studies have revealed that UAE achieved superior yields of baicalin and had the prospects to be applied in the industrial production of baicalin [15]. One of the studies illustrated that the extraction of baicalin was most efficient at temperatures of 60°C, with ethanol concentration of 40% (v/v) [15]. Combinatorial use of HRE with UAE shows higher yield rate and efficiency compared with the use of HRE or UAE alone. Therefore, the combinatorial use of HRE with UAE is also very promising for the commercialized manufacture of natural compounds in the future (e.g. baicalin) [16].

SFE is the process of separating one component (the extractant) from another (the matrix) using supercritical fluids as the extracting solvent. SFE can be used to either strip unwanted material from a product or collect a desired product. Carbon dioxide (CO_2_) is the most widely used supercritical fluid, sometimes modified by co-solvents such as ethanol or methanol. Compared with conventional extraction methods, application of SFE for extraction of flavonoids from herbal medicines was preferable [17]. Different extraction conditions were applied to extract different flavonoids. Modification of extraction conditions may increase the yield of one specific flavonoid, meanwhile decreasing or keeping the yield of another. There are currently ongoing studies aimed to assess the optimal extraction conditions for certain flavonoids. An orthogonal array design (OAD), OA_9_(3^4^), was employed to study the optimal extraction conditions to extract baicalin from herbal medicines using SFE [18]. (Four parameters (modifiers, temperature and pressure of supercritical fluid, and the dynamic extraction time) were studied and optimized by a three-level OAD in which the interactions between the parameters were neglected. Determinations of the extracts were performed by high-performance liquid chromatography. The results showed that selection of the modifier was the main factor in attaining higher yields of baicalin. 1,2-Propanediol-modified supercritical fluid was more effective than 95% ethanol-modified supercritical carbon dioxide or methanol-modified supercritical carbon dioxide for the extraction of baicalin from the solid matrix [18]. Another study illustrated that one of the optimal conditions to extract flavonoids from *Scutellaria baicalensis* using SFE was as follows: supercritical carbon dioxide- MeOH-water (20:2.1:0.9), 50°C and 200 bar [17].

## Anticancer Effect

Baicalin exhibits its anticancer activity against various cancers. The inhibitory effect of baicalin was investigated in two ovarian cancer cell lines (OVCAR-3 and A2780/CP-70) and a normal ovarian cell line (IOSE-364).Baicalin significantly and selectively inhibited the viability of ovarian cancer cells [19]. Meanwhile, it had little adverse side effects on normal ovarian cells [19].

Lacking apoptosis is a hallmark of cancer. Baicalin has been proven to induce apoptosis in human prostate cancer cells [20] and human cervical cancer cells [21]. In baicalin-treated HeLa cells, the expression of Bax, Fas, FasL and Caspase-8 was up-regulated, and the expression of Bcl-2 was down-regulated [21]. It indicated that baicalin triggered apoptosis via activating extrinsic apoptotic pathway.

Baicalin has been reported to retard cancer progression by inhibiting migration, invasion and metastasis of cancer cells. Baicalin suppressed the tumorigenecity of MDA-MB-231 cells by down-regulating the expression of MMP-2, MMP-9, uPA and uPAR through modulating p38MAPK signaling pathway [22].

Angiogenesis is the process of forming new capillary blood vessels from preexisting vasculature leading to neovascularization. Neovascularization is the development of new blood vessels that typically takes place in tissues where circulation has been impaired, either by disease or trauma. Under proper stimulation, endothelial cells begin to form new capillary vessels in the presence of angiogenic factors such as vascular endothelial growth factor (VEGF) and basic fibroblast growth factor (bFGF). Angiogenesis is essential for development and other physiologic conditions. Angiogenesis is now believed to be required for the growth and progression of solid cancers. During solid cancer growth, transformed cells undergo clonal expansion in an avascular state when the expanding lesion is small enough to take in nutrients and to expel metabolic wastes by diffusion [23]. Neovascularization is absolutely required for solid tumor expansion, as the proliferation, as well as metastatic spread, of cancer cells depends on an adequate supply of oxygen and nutrients and the removal of waste products. Cancer cells can stimulate adjacent cells or themselves to release angiogenic factors to promote angiogenesis. Because of the critical dependence of tumor growth and metastasis on angiogenesis, it is suggested that the process of angiogenesis might be a target for therapy. Baicalin decreased the expression of VEGF in two ovarian cancer cell lines (OVCAR-3 and A2780/CP-70) [19]. Liu et al. carried out chicken chorioallantoic membrane (CAM) assay to assess the anti-angiogenic potential of baicalin *in vivo*. The result of CAM assay revealed that baicalin blocked basic fibroblast growth factor-induced angiogenesis in a dose-dependent manner [24].

## Bioavailability

The bioavailability of baicalin after intravenous, intraportal venous, intra-gastric, intra-duodenal, and intra-colonic administration to male rats was studied. Baicalin could not be detected in the hepatic extraction of the rats. The incomplete gastrointestinal absorption seemed to be the main barrier to oral bioavailability and the colon was the main absorption organ of baicalin [25].

The contents of baicalin and its metabolites in the plasma, colon, small intestine, lung, liver, pancreas, kidney, prostate, and in pancreatic tumor were measured in a xenograft animal model. A substantial amount of baicalin (34%–63%) was methylated to oroxylin A and its conjugates in various organs during absorption [26]. Aglycones and conjugates of baicalin were detected in pancreatic tumor and in all tissues investigated except plasma [26]. Plasma contained predominantly conjugates of baicalein, wogonin, and oroxylin A. The result implied that baicalin was potent to be a preventive supplement for pancreatic cancer [26].

Yu et al. found hydrolysis of flavonoids from Baikal skullcap enhanced anticancer activity [27]. Baicalin, baicalein, wogonoside and wogonin are four main flavonoids in Baikal skullcap [28]. The two glycosides (baicalin and wogonoside) can be transformed into their aglycones (baicalein and wogonin), which possess positive anticancer potential. Yu et al. elucidated that catalyzing flavonoids in Baikal skullcap using glycosidase increased the anticancer activities of this herb [27]. Compared with the untransformed control, 8 h of glycosidase catalysis significantly increased anti-proliferative activity on human colorectal and breast cancer cells. The cancer cell growth inhibition was partially mediated by induction of cell cycle arrest at the S-phase and activation of apoptosis [27]. The result suggested that there was a positive correlation between the aglycone content and the anti-proliferative effects. Using glycosidase to catalyze *S. baicalensis* might be a promising approach to modify the bioavailability of baicalin.

## Nanotechnology

**Figure 5:**
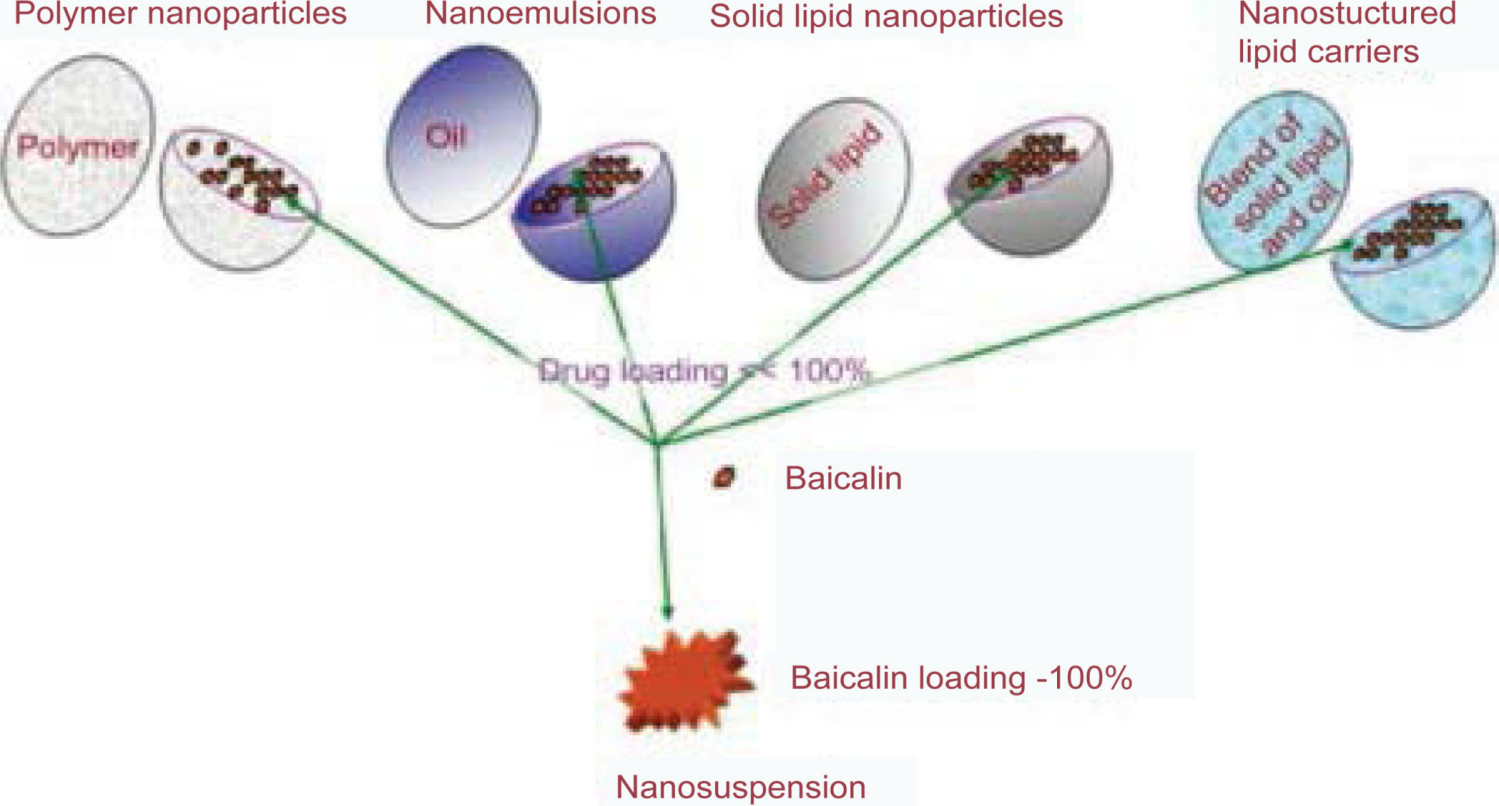
The loading rate of baicalin is much higher in nanosuspension than that in other nanosize delivery systems [29].

Nanotechnology is the manipulation of matter on an atomic or molecular level [29]. Nanonization technology is a promising formulation strategy for poorly water-soluble drugs. Compared with coarse or micronized drug powder, nanodrug particles have appealing advantages, including increased saturation, solubility, and drug-dissolution velocity. So far, various nanonized formulations of baicalin have been used to improve its bioavailability, such as nanoparticles, liposomes, and nanoemulsions. Yue et al. compared the oral bioavailability of regular baicalin crystals (BCN) and baicalin solid nanocrystals (BCN-SNS) in rats. The oral bioavailability of BCN-SNS in rats was remarkably higher than that of BCN or the physical mixture. This result implied that solid nanocrystals might be a good choice for improving the oral bioavailability of poorly soluble baicalin [29]. However, present canonized formulations have disadvantages, such as poor entrapment efficiency and low drug-loading, which led to unsatisfactory results. Recently, nanosuspension (NS) has been successfully used to tackle the formulation problem of poorly soluble drugs. Nanosuspension has remarkable properties, for example, increasing drug solubility and drug-loading capability, which enables its application in the formulation of many poorly soluble compounds (Figure 5) [29].

## Conclusion

Baicalin can be regarded as an alternative treatment for cancers. It elicited anticancer activity and is accumulated in tumor tissues. Unlike the most current cancer treatments, baicalin had little adverse side effects. In this article, we reviewed the common extraction methods used to extract baicalin, the anticancer effect and mechanism of baicalin, the bioavailability of baicalin, as well as the strategies to modify its bioavailability. More animal experiments and clinical trials are required to investigate the anticancer effect and the safety of baicalin and baicalin nanoparticles.
